# Medical Association of Georgia

**Published:** 1866-06

**Authors:** 


*Medical Association of Georgia.*—From the foregoing call of the President, it will be seen that the next meeting of the Association is appointed to be held in Atlanta, on the 21st June, prox. It seems, from Dr. Banks' reason for calling the meeting in Atlanta, that no other place sought it, and that in the position of things, after four years suspension of its functions, the resuscitation is sought, and properly, too, we think, at any point where, from the expression of even a few members, they desire to make preparations for the deliberations of the Association.

We have before alluded to the importance we attach to the transactions of this body. As a means of stimulating members of the profession to investigation on scientific questions, and of promoting the advancement of the medical profession, we think the annual meetings of this body are highly important. While we have no selfish preference for any particular place of meeting, we are pleased in being able to publish the call, and would have been equally so, had it been at any other point. We feel warranted in assuring our professional brethren of the State, that Physicians here, feel a lively interest in the Association, and look to its meetings as a source of advancement in medical science, and a blessing to the objects of medical skill.

The new feature in the programme, adopted at last meeting—that of calling for prize essays—we think is a good one. We have no intimation of any intending to produce an essay for this purpose, but as it is understood that the prizes will be ready for the successful competitors, we suppose essays for this purpose will be presented.

